# Longer Hydroxyurea Administration Prior to Imatinib Mesylate is Risk Factor for Unsuccessful Major Molecular Response in Chronic-Phase Chronic Myeloid Leukemia: Possibility of P-Glycoprotein Role

**DOI:** 10.31557/APJCP.2019.20.12.3689

**Published:** 2019

**Authors:** Ikhwan Rinaldi, Ary Harryanto Reksodiputro, Sri Widia Jusman, Alida Harahap, Rianto Setiabudy, Septelia Inawati Wanandi, Karmel Tambunan, Catharina Suharti

**Affiliations:** 1 *Department of Internal Medicine Division of Hematology and Medical Oncology, *; 3 *Department of Clinical Pathology, *; 4 *Department of Clinical Pharmacology, Faculty of Medicine Universitas Indonesia-Dr. Cipto Mangunkusumo Hospital, *; 2 *Department of Biochemistry, Faculty of Medicine Universitas Indonesia, Jakarta, *; 5 *Department of Internal Medicine Division of Hematology and Medical Oncology, Faculty of Medicine Universitas Diponegoro-Dr. Karyadi Hospital, Semarang, Indonesia. *

**Keywords:** Chronic myeloid leukemia, major molecular response, hydroxyurea, imatinib mesylate, P-glycoprotein

## Abstract

**Objective:**

This study aimed to identify the association between duration of HU administration prior to IM treatment and MMR achievement in chronic-phase CML while evaluating the role of MDA, HIF-1α and P-gp.

**Methods:**

The study was conducted at Dr. Cipto Mangunkusumo National General Hospital and Dharmais Cancer Hospital, Jakarta using retrospective cohort design to analyse the association between the duration of HU before IM and its MMR achievement and cross-sectional design to analyse the association between MDA, HIF-1α and P-gp expressions with MMR achievement. Main subjects were chronic-phase CML patients treated by HU prior to IM for **≥** 12 months and HU only. The subjects were divided into four main groups: (1) chronic-phase CML patients treated with HU ≤ 6 months + IM ≥ 12 months and (2) HU > 6 months + IM ≥ 12 months (3) HU only (≤ 6 months), (4) HU only ( >6 months). Subjects were obtained from January 2015 to May 2016. Data were gathered through history taking, physical examination, medical record evaluation, and blood sample analysis. Bivariate analysis was conducted using chi square, independent T-test, and Mann-Whitney according to the variables.

**Results:**

Administration of HU for more than 6 months prior to IM was associated with unsuccessful MMR achievement (RR 1.60; 95%CI 1.29-2.00). MDA level, HIF-1α, P-glycoprotein expression were not associated with MMR achievement but the mean MDA level (0.63±0.31 vs 0.75±0.41 p=0.461) and median P-glycoprotein expressions **{**16,92 (0,04 – 43,86) vs. 5,15 (0,02–39,64); p=0.311**}** were found to be higher in patients receiving HU for > 6 months group than in HU ≤ 6 months group consecutively.

**Conclusion:**

Administration of HU for more than 6 months prior to IM was associated with unsuccessful MMR achievement in chronic-phase CML. The study suggested that P-glycoprotein overexpression as the predictor for unsuccessful MMR achievement.

## Introduction

Chronic myeloid leukemia (CML) is a myeloproliferative disorder marked by leukocytosis and the presence of myeloblast in various stages in the peripheral blood due to persistent activation of BCR-ABL gene, which encodes proteins involved in activation of tyrosine kinase transduction pathway (Hochhaus et al., 2009; Rao et al.,2010; Radich et al., 2016; Druker et al., 2001). CML can evolve from chronic phase to accelerated and blastic crisis in 3-5 year time until death, usually 3-6 month after blastic crisis (Baccarani et al., 2012; Shet et al., 2002; Radich et al., 2016; Druker et al., 2001).

Tyrosine kinase inhibitor (TKI) such as imatinib mesylate (IM) is now standard of treatment for CML, targeting its pathogenesis. This treatment aims to help patients have major molecular response (MMR), defines as BCR-ABL1 transcription ≤0.1% international scale after 12 months of treatment (Radich et al., 2016; Baccarani et al., 2012). MMR number according to International Randomized IFN vs. STI571 Study (IRIS) was 39%, MMR in Asia was 42% (Than et al., 2012; Radich et al., 2016) MMR number in Indonesia is still unknown, but Reksodiputro et al., (2010) shown that complete hematologic response (CHR), defines as normal hematologic parameter after 3 months of IM treatment, in Indonesia was 74%, while IRIS study shown 95.3.% (O’Brien et al., 2003). Unpublished study by Rinaldi (2012) also shown that subjects given hydroxyurea (HU) ≥ 6 months had less CHR percentage than HU < 6 months (35.7% vs. 70%; p < 0.05). Thus, it is implied that HU duration > 6 months associated with MMR.

HU administration for ≥ 6 months increased reactive oxygen species (ROS) production (Sakano et al., 2001). ROS production is induced by BCR-ABL protein through PI3K/AKT/mTOR pathway (Sattler et al., 2000; Kim et al., 2005). ROS restricts HIF-1α degradation which is part of hypoxia inducible factor-1 (HIF-1). These increase expression of HIF-1α dan HIF-1 which then induce MDR1 gene to overly express P-glycoprotein (*P-gp*), an efflux protein that can reduce (IM) effectivity (Semenza, 2001; Nagy, 2011; Ullah, 2008). In vitro study by Mahon et al., (2003) shown that overexpression of P-gp reduced imatinib efficacy in cells with BCR-ABL positive. IM treatment was also found increased P-gp expression only in *P-gp* positive cells, not in the negative ones (Vasconcelos et al., 2011).

This study aimed to know the association between duration of HU administration prior to IM treatment and MMR achievement in chronic-phase CML, as well as role the of ROS, HIF-1α and P-gp in the process. ROS was represented by the level of malondialdehyde (MDA) as the result of ROS reaction with lipid inside cells (Sattler et al., 2000; Kim et al., 2005).

## Materials and Methods

*Study design and subjects*

The study used retrospective cohort study design to analyze the association between the duration of HU administration prior to IM treatment and its MMR achievement. At the same time, cross sectional study was also used to analyze the association between *MDA* plasma levels, *HIF-1α* and *P-gp* expression after IM treatment and MMR achievement.

Subjects were CML chronic phase patients who were diagnosed from clinical, hematological, and molecular findings (positive *BCR-ABL/ABL*) and were treated with HU only or HU for at least two weeks, and then continued with IM for ≥ 12 months. We used HU only to estimate the roles of *MDA* level, *HIF-1α*, and *P-gp* expression in *MMR* achievement after IM treatment and used the minimum duration of two weeks for HU treatment prior to IM due to clinical consideration in our hospital. Usually, HU was administered prior to IM treatment for at least two weeks during the waiting period for *BCR-ABL/ABL *examination result, especially in patients who manifested as hyperleukocytosis. Patients who received IM for more than 400 mg were excluded. Subjects were selected consecutively. 

The study was done at the Outpatient Clinic of Hematology and Clinical Oncology in Dr. Cipto Mangunkusumo Hospital, and Dharmais Cancer Hospital, Jakarta between January 2015 and May 2016. Minimal sample size was calculated using sample size calculation for retrospective cohort and cross-sectional design. 

*Data collection*

Data were collected from history taking, physical examination, medical records, and blood samples (15 mL). Baseline characteristics, duration of HU and IM treatment, complete blood count, *BCR-ABL/ABL *ratio (*MMR* achievement), MDA plasma level, *HIF-1α *expression, and *P-gp* expression were collected at the same time. Blood samples from the subjects were obtained from the patients who were already treated with with HU only or HU followed by IM for **≥** 12 months. In the same time, they were analysed to identify the *BCR-ABL* ratio, MDA plasma level, *HIF-1α* and P-glycprotein expression. Identification of MDA plasma level and *HIF-1α* expression were conducted at the Laboratory of Biochemistry, Faculty of Medicine, Universitas Indonesia, identification of P-glycoprotein expression was conducted at the Laboratory of Clinical Pharmacology, Faculty of Medicine, Universitas Indonesia, and the examination of *BCR-ABL/ABL* ratio was performed at the Laboratory of Clinical Pathology Dharmais Cancer Hospital.

The examination of MDA plasma level was performed using thiobarbituric acid reaction substances (TBARS) technique (Ahmad et al., 2008b). *HIF-1α* was evaluated by enzymed-linked immunosorbent assay (ELISA) using a kit from Abcam with a catalogue number of ab171577.

The examination of P-gp expression was carried out using quantitative real-time reverse transcription polymerase chain reaction (qRT-PCR) (Louisa et al., 2014). RNA was isolated from the samples using Tripune Isolation Reagen according to the manufacturer protocols. The quantity and purity of RNA were then determined by measuring 260/280 absorbance using NanoDrop™ spectrophotometer (Thermo Fisher Scientific™). The RNA produced was then used for quantitative examination of P-gp expression. The examination of P-gp expression was continued by qRT-PCR technique using LightCycler^®^ RNA Master SYBR Green I kit and LightCycler^®^ 2.0 Instrument according to the manufacturer’s instruction (Roche Molecular Systems, Inc). The transporter primer utilized in the study was the primer that had been used before. The primer for* P-gp *transporter was F: CCATCATTGCAATAGCAGG; *P-gp *R: GTTCAAAACTTCTG CTGGTCA.23 The relative changes in mRNA expression of P-gp level were measured using Livak’s method (Livak and Schmittgen, 2001).

Quantitative examination of *BCR-ABL* was performed by qRT-PCR (Foroni et al., 2011). RNA was isolated from the samples and subsequently reverse transcription. Amplification were carried out using specific target primer of *p210 BCR-ABL* and region gene *ABL* as the internal control using Accupower® *BCR-ABL *quantitative RT-PCR kit (Bioneer Corporation, catalogue number CML-1111). Quantitative result was obtained through relative comparison between the level of gene expression *p210 BCR-ABL* and *ABL* gene against the standard curve. The results of *p210 BCR-ABL* were presented as a percentage according to the international scale (Foroni et al., 2011).

*Statistical methods*

Baseline characteristics of the subjects were described as mean or median for numerical data and percentage for categorical data. Duration of HU administration was categorized into two groups: HU ≤ 6 months dan > 6 months since IRIS study was also included subjects treated with HU for 6 months before IM treatment (Hochhaus et al., 2009). Duration of IM administration after HU was ≥ 12 months since previous studies shown more subjects treated by IM > 12 months had achieved MMR and did not progress to acceleration and blastic phase (O’Brien et al., 2014). Main subjects were divided into four main groups: subjects who were administered with (1) HU for >6 months + IM for ≥ 12 months and (2) HU for > 6 months + IM for ≥ 12 months (3) HU only (≤ 6 months), and (4) HU only (>6 months). Successful MMR achievement was defined as ratio *BCR-ABL/ABL* ≤ 0.1% examined by qRT-PCR technique after IM treatment ≥ 12 months (Baccarani et al., 2013).

Statistical analyses were performed using IBM Statistical Package for the Social Sciences (SPSS)^®^ for Windows version 20.0. In all analysis, p value ≤0.05 for the two-tailed test was considered significant. Association between duration of HU administration prior to IM treatment and MMR achievement was carried out using Chi Square test. Role of *MDA* plasma level, *BCR-ABL/ABL* ratio, HIF-1α expression, and *P-gp* expression in *MMR* achievement were analyzed using independent T-test or Mann Whitney, depend on distribution of the numerical data.

*Ethics statement*

Ethical clearance of this study was released by Ethics Committee of Faculty of Medicine, Universitas Indonesia – Dr. Cipto Mangunkusumo Hospital No. 139/UN2.F1/ETIK/2015. Permission to conduct the study was given by Dr. Cipto Mangunkusumo Hospital and Dharmais Cancer Hospital. Written consents were obtained from the subjects after being informed about the study protocol.

## Results

A total of 123 subjects were included in this study. The median age was 40 years. Male were the majority of the subjects). Highest successful *MMR* achievement was reached by HU ≤ 6 months + IM ≥ 12 months group (41.2%), while HU > 6 months + IM ≥ 12 months group had the lowest *MMR* achievement (5.9%). The duration of both HU treatment and IM treatment in our subjects had wide ranges (0-168 months for HU treatment and 0-72 months to for IM treatment). Subject baseline characteristics can be viewed in Table 1. 

Duration of HU administration prior to IM treatment was associated with *MMR* achievement (p = 0.001). Duration of HU administration more than 6 months before IM treatment was a risk factor of unsuccessful *MMR* (RR 1.60; 95%CI 1.29-2.00). The median of *MDA *plasma level, *HIF-1α*, and *P-gp* expression after IM treatment were not significantly different between *MMR *achievement groups. However, higher MDA level and *P-gp* expression were found in unsuccessful MMR group compared to the successful one (Table 2).

*MDA* level, and *P-gp* expression in HU for > 6 months group were found to be significantly higher than in HU for ≤ 6 months group. *BCR-ABL/ABL* ratio was also found to be lower in HU for >6 months group as shown in Table 3.

**Table 1:** Subject characteristics

Variables	All subjects n = 123
Age (years), median (minimum-maximum)	40.0 (18.0–79.0)
Gender, n (%)	
Female	58 (47.2)
Male	65 (52.8)
Subject groups according to treatment, n (%)	
HU ≤ 6 months	13 (6.3)
HU > 6 months	8 (3.9)
HU ≤ 6 months + IM ≥ 12 months	68 (33.2)
HU > 6 months + IM ≥ 12 months	34 (16.6)
Hematology	
Haemoglobin (g/dL) (mean)	
HU ≤ 6 months	10.80 (8.10–13.40)
HU > 6 months	13.25 (6.90–14.50)
HU ≤ 6 months + IM ≥ 12 months	12.00 (3.20–22.90)
HU > 6 months + IM ≥ 12 months	11.60 (8.40 –18.20)
Leukocytes (/μL) median (min-max)	
HU ≤ 6 months	29,500 (3,400 – 226,000)
HU > 6 months	15,400 (4,600–110,700)
HU ≤ 6 months + IM ≥ 12 months	6,300 (2,500 – 75,130)
HU > 6 months + IM ≥ 12 months	8,850 (2,300– 100,500)
Platelet (/μL) median (min-max)	
HU ≤ 6 months	325,000 (109,000– 1,090,000)
HU > 6 months	410,500 (149,000 –1,240,000)
HU ≤ 6 months + IM ≥ 12 months	236,500 (85,000– 1,230,000)
HU > 6 months + IM ≥ 12 months	222,500 (14,400 – 1,220,000)
Basophils (%) median (min-max)	
HU ≤ 6 months	5.0 (2.0 –12.0)
HU > 6 months	3.0 (2.0–10.0)
HU ≤ 6 months + IM ≥ 12 months	0.0 (0.0–47.0)
HU > 6 months + IM ≥ 12 months	0.0 (0.0–19.0)
Blast cells (%)	
HU ≤ 6 months	1.0 (0.0–11.0)
HU > 6 months	0.5 (0.0–18.0)
HU ≤ 6 months + IM ≥ 12 months	0.0 (0.0–2.0)
HU > 6 months + IM ≥ 12 months	0.0 (0.0–16.0)
Duration of treatment (months), median (min-max)	
HU	2 (0–168)
IM	13 (0–72)

Abbreviations, HU, Hydroxyurea; MMR, Major Molecular Response; IM, Imatinib mesylate; min, minimum; max, maximum

**Table 2:** Association between Duration of HU Administration Prior to IM Treatment (≥ 12 months), *MDA*, *HIF-1α*, *P-gp* Level and *MMR* Achievement in Chronic-phase Chronic Myeloid Leukemia

		MMR Achievement				RR	p value
		Unsuccessful n (%)	Successful n (%)	Unsuccessful median (min-max)	Successful median (min-max)	(95% CI)	
Duration of HU treatment prior to IM (months) n (%)	> 6 ≤ 6	32 (94.1) 40 (58.8)	2 (5.9) 28 (41.2)			1.60 (1.29-2.00)	0.001†
*MDA* level (nmol/mL)				0.66 (0.08–1.12)	0.51 (0.09–1.11)	Not available	0.134*
*HIF-1α* level (ng/mg protein)				0.03 (0.01–0.27)	0.03 (0.01–0.27)	Not available	0.383*
*P-gp* expressions (*P-gp/β*-actin ratio)				0.10 (0.01–88.85)	0.04 (0.01–103.85)	Not available	0.161*

† Chi square test, two sided P.value<0.05; * Mann-Whitney test, two sided P. value>0.05; Abbreviations, HU, Hydroxyurea; MMR, Major Molecular Response; IM, Imatinib mesylate; MDA, malondialdehyde; HIF, Hypoxia inducible factor; P-gp, P-glycoprotein; min, minimum; max, maximum

**Table 3.** Differences of *BCR-ABL/ABL* ratio, *MDA* level, *HIF-1α* level, and* P-gp *Expression between Duration of HU only Administration Groups

	HU ≤ 6 months (n = 13)	HU > 6 months (n = 8)	p value
*BCR-ABL/ABL* ratio (%)	66.27 ± 49.09	52.44 ± 41.46	0.515*
*MDA* level (nmol/mL)	0.63 ± 0.31	0.75 ± 0.41	0.461*
*HIF-1α* level (ng/mg protein)	0.03 ± 0.02	0.03 ± 0.03	0.491*
*P-gp* expression (*P-gp/β*-actin ratio)	5.15 (0.02–39.64)	16.92 (0.04–43.86)	0.311**

*Unpaired T test **Mann-Whitney test, two sided P. value<0.05; Abbreviations, HU, Hydroxyurea; MMR, Major Molecular Response; IM, Imatinib mesylate; MDA, malondialdehyde; HIF, Hypoxia inducible factor; P-gp, P-glycoprotein; min, minimum; max, maximum

## Discussion

Ideally, *MDA* level, *HIF-1α*, and* P-gp* expression should be measured after the subjects received HU for < 6 months or > 6 months before followed by IM administration, and measurement of MMR achievement (*BCR-ABL *ratio) is done after the subjects received IM for 12 months. Unfortunately, this will require a long period of time and difficult to be conducted, therefore the study was conducted using cohort retrospective design to determine the association between the duration of HU administration prior to IM treatment **≥** 12 months and its MMR achievement. *MDA* level, *HIF-1α*, and *P-gp *expressions were measured after HU was administered to the subjects which later was divided into two groups based on its duration, either for < 6 months or > 6 months. The results indirectly identified the roles of MDA levels, *HIF-1α*, and *P-gp* expressions which acted as predictors for MMR achievement after IM treatment for **≥** 12 months. 

The practice of using hydroxyurea as cytoreductive agent prior to imatinib mesylate is still commonly done, especially in Indonesia. This practice was also done in International Randomized IFN vs. STI571 (IRIS) Study for subjects who had leukocyte count >20,000 cells/µL. IRIS study had limited the duration of hydroxyurea prior to imatinib mesylate by 6 months, but the rationale was lacking (O’Brien et al., 2003). Moreover, we had a lot of patients who had been given hydroxyurea more than 6 months prior to IM treatment due to financial issue (IM was not covered by national health insurance until, 2015). In our preliminary study, subjects given hydroxyurea (HU) ≥ 6 months had worse hematologic response than HU < 6 months (Rinaldi, 2012). Thus, our study investigated the association between duration of hydroxyurea treatment prior to imatinib mesylate treatment and major molecular response in chronic-phase chronic myeloid leukemia.

Subjects’ characteristics were different from other studies in developed, Western countries. The median of age found in this study (40 years old) was similar as previous studies in Asia (36-55 years old), which were 2 decades younger than in developed countries with higher gross domestic product (GDP) (Au et al., 2009; Mendizabal et al., 2016). Different carcinogenic exposure and genetical vulnerability between Asian and Western population could be the cause of this differences, despite the need of more studies to prove it (Mendizabal et al., 2016). The different study population between our and other studies had added our study importance especially for Asian countries population.

Our study showed *MMR* achievement from HU only for ≤ 6 months group was 66.3%, and HU only for > 6 months group was 52.4%. This data showed that the achievement of MMR was higher in the group receiving HU for less than 6 months, the finding was also supported with *MMR* achievements from groups receiving HU followed by IM with HU ≤ 6 months + IM ≥ 12 months and HU > 6 months + IM ≥ 12 months (41.2% vs 5.9%, p=0.001 consecutively). We found that the duration of HU administration > 6 months prior to IM treatment was associated with less successful MMR achievement (RR=1.60; 95% CI 1.2-2.00; p=0.001). This finding provided the rationale for limiting the duration of hydroxyurea prior to imatinib mesylate by 6 months, such as done by IRIS study (O’Brien et al., 2003).

This association is explained by the role of reactive oxygen species (ROS) generated by hydroxyurea. ROS induces signal transduction to synthesize *HIF-1α* through activation of *PI3K/AKT/mTOR* pathway (Naughton et al., 2009). ROS also inhibits *HIF-1α* degradation, resulting dimerization with HIF-1α in nucleus. The dimerization activates several genes consist of hypoxia-response element (HRE), one of them expressing *P-gp*, an efflux protein that can reduce (IM) effectivity.(Semenza, 2001; Nagy, 2011; Ullah, 2008) Longer duration of HU administration causes higher amount of ROS production, resulting overexpression of *P-gp*. Thus, ROS (expressed by MDA level), *HIF-1α* and *P-gp* are thought to have causal role in the association between the duration of HU administration (> 6 months) before IM treatment and unsuccessful MMR achievement. 

In our study, MDA level mean and *P-gp* expression median in HU > 6 months group were higher than in HU ≤ 6 months group 0.75±0.41 vs 0.63±0.31 p=0.461 and {16,92 (0,04 – 43,86) vs. 5,15 (0,02–39,64)} consecutively , though they were statistically insignificant due to the small sample size obtained. High expression of *P-gp* in HU only > 6 months group represented the condition before IM administration, as shown by the results from Stromskaya et al., (2008) and Vasconcelos et al., (2011). Both studies showed that P-gp expressions were higher in subjects whose *P-gp* expression was already high prior to IM administration. 

Unsuccessful *MMR* achievement from the group receiving HU for > 6 months + IM ≥ 12 months was supported with the high P-gp expression finding. This phenomenon could be explained by the role of P-gp, an efflux protein of IM proposed to be associated with imatinib resistance in CML patients (Mahon et al., 2003; Peng et al., 2012). We hypothesized that in subjects with high P-gp expression prior to IM treatment such as patients with duration of HU treatment more than 6 months, their blast cells could efflux IM, resulting unsuccessful MMR achievement. Conversely, the leukemic cells in subjects with low P-gp expression prior to IM treatment, such as patients with duration of HU treatment up to 6 months, could not efflux IM, resulting successful *MMR* achievement. Thus, we suggested that *P-gp* expression has a role in achieving* MMR*. 

However, our study could not find statistically significant relationship between *P-gp* expression and MMR achievement due to our limitated sample size, similar with a study by Malhotra et al., (2015) which also had small sample size. There were few subjects in our study whose* P-gp* expression were high but achieved *MMR*, confounding the statistical analysis. We hypothesized that even these patients had achieved *MMR*, the remaining blast cells were highly expressed *P-gp*, thus in risk of having disease progression in the future. Our hypothesis was supported by Galimberti et al., (2005) who observed 3 chronic-phase CML patients which were progressing after remission in the early period of therapy, all of which showed increased *P-gp* expression 3 months earlier. 

Although there were slight increases of *MDA* level between *MMR* achievement groups but these increases were not significant. These results could be instigated by small sample size and the role of IM treatment. *MDA* plasma level was a product resulted from induction of *BCR-ABL* gene through signal transduction in *PI3K/AKT/mTOR* pathway, while IM was reducing the *BCR-ABL/ABL* ratio. Thus, low *MDA* level is the direct consequence of low *BCR-ABL/ABL* ratio caused by IM treatment (Sattler et al., 2000; Kim et al., 2005). Moreover, several studies shown different MDA level between different phase of CML (Petrola et al., 2012; Ahmad et al., 2008a, 2008b). All subjects included in this study was at chronic phase, so there were no differences of *MDA *level between groups evaluated.

For* HIF-1α* expression, micro-environment of bone marrow is in hypoxic state which aim to renewal of hematological stem cells physiologically (Zhang et al., 2012). This hypoxic environment also preserves leukemic stem cells due to increased activity of *HIF-1α*. Moreover, IM treatment increases *HIF-1α* activation through MAPK pathway in leukemia progenitor cells (Chu et al., 2004). Ng et al., (2014) presented an increasement of progenitor cells colony of CML twice in hypoxic state, and 2-5 times more in IM treatment. Therefore,* HIF-1α *expression is maintained in any condition of chronic phase CML patients due to physiologically hypoxic state and the effect of IM treatment towards bone marrow to preserve both leukemic and healthy stem cells. Despite there was an increase of MDA level between the group achieved MMR and the group who did not, there was no increase for HIF-1α level on both groups (0.03 (0,01-0,27) vs 0,03 (0,01-0,27))

*Study Limitation*

There were several limitations of this study. Our study could not evaluate the role of *MDA*, *HIF-1 HIF-1α*, *P-gp*, and *BCR-ABL/ABL* ratio in ideal manner as prospective cohort study will be. Ideally, evaluation of MDA level, *HIF-1α*, *P-gp* expression are done before and after each treatment (by HU and IM), while our study could only evaluate all of the factors once after treatment. 

We did not restrict the maximum duration of IM and HU treatment (72 and 168 months, respectively), thus selection bias might be present. 

Further prospective cohort studies with bigger sample size and limited duration of HU and IM are needed to prove the role of *BCR-ABL/ABL* ratio, *MDA*, *HIF-1α*, and P-gp on *MMR* achievement in chronic phase CML patient. 

Duration of hydroxyurea (HU) administration more than 6 months prior to Imatinib Mesylate (IM) treatment was associated with unsuccessful major molecular response (*MMR*). Therefore, the duration of hydroxyurea administration prior to imatinib mesylate treatment should be limited until 6 months only. P-glycoprotein (*P-gp*) was overexpressed in group with longer HU duration prior to IM treatment. We suggested that there was some role of P-glycoprotein overexpression in unsuccessful *MMR *achievement, despite the lack of significant association statistically. Meanwhile, *MDA* and* HIF-1α *were not related to duration of HU prior to IM treatment and *MMR *achievement. This study could be used as reference in treating chronic phase CML patients with HU and IM in daily practice and to do follow-up studies evaluating *P-gp* overexpression as a predictor of treatment outcome.
